# Cardiac involvement in Behçet’s syndrome: findings from a clinically driven cardiological evaluation

**DOI:** 10.1093/rheumatology/keag292

**Published:** 2026-07-02

**Authors:** Alessandra Bettiol, Federica Bello, Ruggero Mazzotta, Irene Mattioli, Giacomo Bagni, Edoardo Biancalana, Maria Canfora, Mariapaola Lisi, Danilo Malandrino, Miki Palmerini, Maurizio Pieroni, Maria Romanelli, Maria Letizia Urban, Savino Sciascia, Domenico Prisco, Elena Silvestri, Iacopo Olivotto, Giacomo Emmi

**Affiliations:** Department of Experimental and Clinical Biomedical Sciences “Mario Serio”, University of Florence, Florence, Italy; Department of Experimental and Clinical Medicine, University of Florence, Florence, Italy; Department of Experimental and Clinical Medicine, University of Florence, Florence, Italy; Arrhythmia and Electrophysiology Unit, Careggi University Hospital, Florence, Italy; Department of Experimental and Clinical Medicine, University of Florence, Florence, Italy; Department of Clinical and Biological Sciences, University of Torino, Torino, Italy; Department of Experimental and Clinical Medicine, University of Florence, Florence, Italy; Department of Experimental and Clinical Medicine, University of Florence, Florence, Italy; Department of Experimental and Clinical Medicine, University of Florence, Florence, Italy; Hospital Pharmacy Unit, Department of Drug and Medical Device, Careggi University Hospital, Florence, Italy; Department of Experimental and Clinical Medicine, University of Florence, Florence, Italy; Department of Experimental and Clinical Medicine, University of Florence, Florence, Italy; Department of Experimental and Clinical Medicine, University of Florence, Florence, Italy; Cardiomyopathy Unit, Careggi University Hospital, Florence, Italy; Department of Experimental and Clinical Biomedical Sciences “Mario Serio”, University of Florence, Florence, Italy; Department of Medical, Surgery and Health Sciences, University of Trieste, Trieste, Italy; Department of Clinical and Biological Sciences, University of Torino, Torino, Italy; Department of Experimental and Clinical Medicine, University of Florence, Florence, Italy; Department of Experimental and Clinical Medicine, University of Florence, Florence, Italy; Department of Experimental and Clinical Medicine, University of Florence, Florence, Italy; Cardiology Unit, Meyer Children’s Hospital IRCCS, Florence, Italy; Department of Medical, Surgery and Health Sciences, University of Trieste, Trieste, Italy; Centre for Inflammatory Diseases, Monash University Department of Medicine, Monash Medical Centre, Melbourne, Australia; Immunology and Allergy Service, CHUV, Lausanne, Switzerland

**Keywords:** arrhythmias, Behçet’s syndrome, cardiac involvement, cardiovascular risk, ischemic heart disease, pericarditis, retrospective cohort, systemic vasculitis

## Abstract

**Objectives:**

To characterize prevalence, patterns, and clinical correlates of cardiac involvement in a cohort of patients with Behçet’s syndrome (BS) undergoing clinically driven cardiological assessment.

**Methods:**

This retrospective study included adult patients with BS, followed at a tertiary referral centre (Florence, Italy), who underwent clinically driven cardiological evaluation. Clinical, demographic and therapeutic characteristics were compared in patients with and without evidence of cardiac involvement. Timing, type of cardiac involvement, disease activity, cardiovascular risk factors and treatments were analysed.

**Results:**

Among 312 patients with BS, 90 underwent cardiological assessment and 49 had confirmed cardiac involvement. Arrhythmias were the most frequent manifestation (*n* = 15), followed by pericarditis (*n* = 12) and ischaemic heart disease (*n* = 8). Cardiac involvement occurred a median of 6.1 (2.5–8.7) years after BS diagnosis, although in a proportion of patients it preceded or coincided with diagnosis. Most patients had active systemic disease at the time of the cardiac involvement (93.9%) and were receiving immunosuppressant treatment (84%). Over 80% had at least one traditional cardiovascular risk factor, yet <40% were receiving cardiovascular primary prevention therapy before the first event. Male patients more frequently experienced ischaemic manifestations, while arrhythmias were more common in females. No cardiac-related deaths were observed.

**Conclusion:**

Cardiac involvement in BS is more common than traditionally reported when actively investigated. Cardiac involvement frequently occurs during active disease and in patients with a high burden of cardiovascular risk factors but suboptimal preventive therapy. These findings support the need for integrated cardio-rheumatologic management in BS.

Rheumatology key messages

- Cardiac involvement is more frequent in Behçet’s syndrome than previously reported when clinically driven cardiological evaluation is performed.
- Cardiac involvement mainly occurs in patients with active systemic disease, with arrhythmias and pericarditis being the most frequent.
- Cardiac involvement frequently occurs despite immunosuppressive therapy and in patients with high cardiovascular risk burden.

## Introduction

Behçet’s syndrome (BS) is a chronic, relapsing, multisystem vasculitis characterized by recurrent oral and genital ulcers and ocular inflammation, frequently accompanied by cutaneous, articular, vascular, neurological and gastrointestinal manifestations [1]. Cardiac involvement in BS has traditionally been considered rare, with a reported prevalence around 5–8% in clinical series [2, 3]. However, this frequency may be underestimated due to subclinical presentations and variability in diagnostic approaches. Indeed, a post-mortem study from a Japanese autopsy registry reported cardiac lesions in 16% of patients with BS [4], and more recent imaging studies have demonstrated evidence of subclinical cardiac abnormalities, particularly through cardiac MRI [5].

Cardiac involvement in BS is known to encompass a heterogeneous spectrum of manifestations, affecting the pericardium, myocardium, endocardium, coronary arteries and cardiac valves [6]. These manifestations reflect distinct but interconnected pathogenic mechanisms that mirror the systemic pathophysiology of the disease [7]. Direct inflammatory infiltration of cardiac tissues has been documented, particularly in cases of intracardiac thrombosis, endomyocardial fibrosis, valvular inflammation and coronary artery involvement [8, 9]. Concurrently, systemic immune dysregulation promotes endothelial activation and dysfunction, leading to increased vascular permeability, leucocyte adhesion, intimal proliferation and luminal narrowing [10]. Persistent cytokine overexpression and immune-cell infiltration within myocardial and pericardial tissues may result in inflammatory cardiomyopathy or pericarditis. Moreover, neutrophil hyperactivity and enhanced oxidative stress contribute to a prothrombotic state, which is involved in the pathogenesis of intracardiac and coronary thrombotic events [11–14]. Cardiac involvement has been associated with a 5-year mortality of ∼16% in BS [2], with a mortality rate exceeding vascular as well as CNS involvement. Despite its clinical relevance, cardiac involvement in BS remains insufficiently characterized in large, well-defined cohorts.

In this retrospective clinically driven cohort study, we reviewed medical records of all patients with BS who underwent cardiological assessment, either for a scheduled follow-up or due to symptoms suggestive of cardiac involvement. We compared demographic, clinical and therapeutic characteristics in patients with *vs* those without evidence of cardiac involvement, and described the patterns and timing of cardiac involvement, and their association with disease activity, treatment exposure and traditional cardiovascular risk factors, with the goal of informing clinicians about this underrecognized but clinically relevant manifestation of BS.

## Patients and methods

We conducted a retrospective, non-interventional study including adult patients with BS, followed at the Behçet Center of the Careggi University Hospital, Florence, Italy, during the period 1 November 2011–31 October 2024.

Patients were eligible for inclusion if they fulfilled the International Criteria for Behçet’s Disease and/or the International Study Group criteria for BS, and had undergone a cardiological evaluation as part of routine clinical practice, either for a scheduled follow-up or due to symptoms suggestive of cardiac involvement. Routine cardiological assessment included clinical exam, ECG and 2D-echocardiography; when clinically indicated, second-level investigations included cardiac MRI and coronary angiography.

Cardiac involvement was defined as the evidence of inflammatory myocardial, pericardial or endocardial disease, and/or evidence of intracardiac or coronary thrombosis.

Medical records of these patients were systematically reviewed to retrieve demographic, clinical, diagnostic and therapeutic data, which were compared in patients with *vs* without evidence of cardiac involvement at cardiological assessment. Information on disease activity at time of cardiac event was also retrieved; specifically, disease activity was assessed by means of the Behçet’s Disease Current Activity Form (BDCAF), and active disease was defined by a BDCAF ≥1.

Data were retrieved for the entire disease course, from BS diagnosis to the occurrence of the first cardiac event, as well as at the time of the first cardiac event and at the time of the first cardiac relapse, when applicable.

When information in the medical records was incomplete for certain variables (e.g. traditional cardiovascular risk factors), patients were contacted by phone to obtain the missing data.

The study was conducted in accordance with the Declaration of Helsinki and was approved by the Ethics Committee of Area Vasta Centro (reference no. 2024–096; ID 21395_BIO). Written informed consent was obtained from all participants.

### Statistical analysis

Continuous variables are presented as median values with interquartile range (IQR), while categorical variables are reported as absolute frequencies and percentages. Statistical analyses were performed using STATA SE version 18 (StataCorp, College Station, TX, USA). Inferential analyses were conducted to compare demographic, clinical and therapeutic features in patients with *vs* those without evidence of cardiac involvement at cardiological assessment, by using Fisher’s exact test for dichotomic variables and the Mann–Whitney test with exact p-values for continuous variables.

## Results

**Figure 1:**
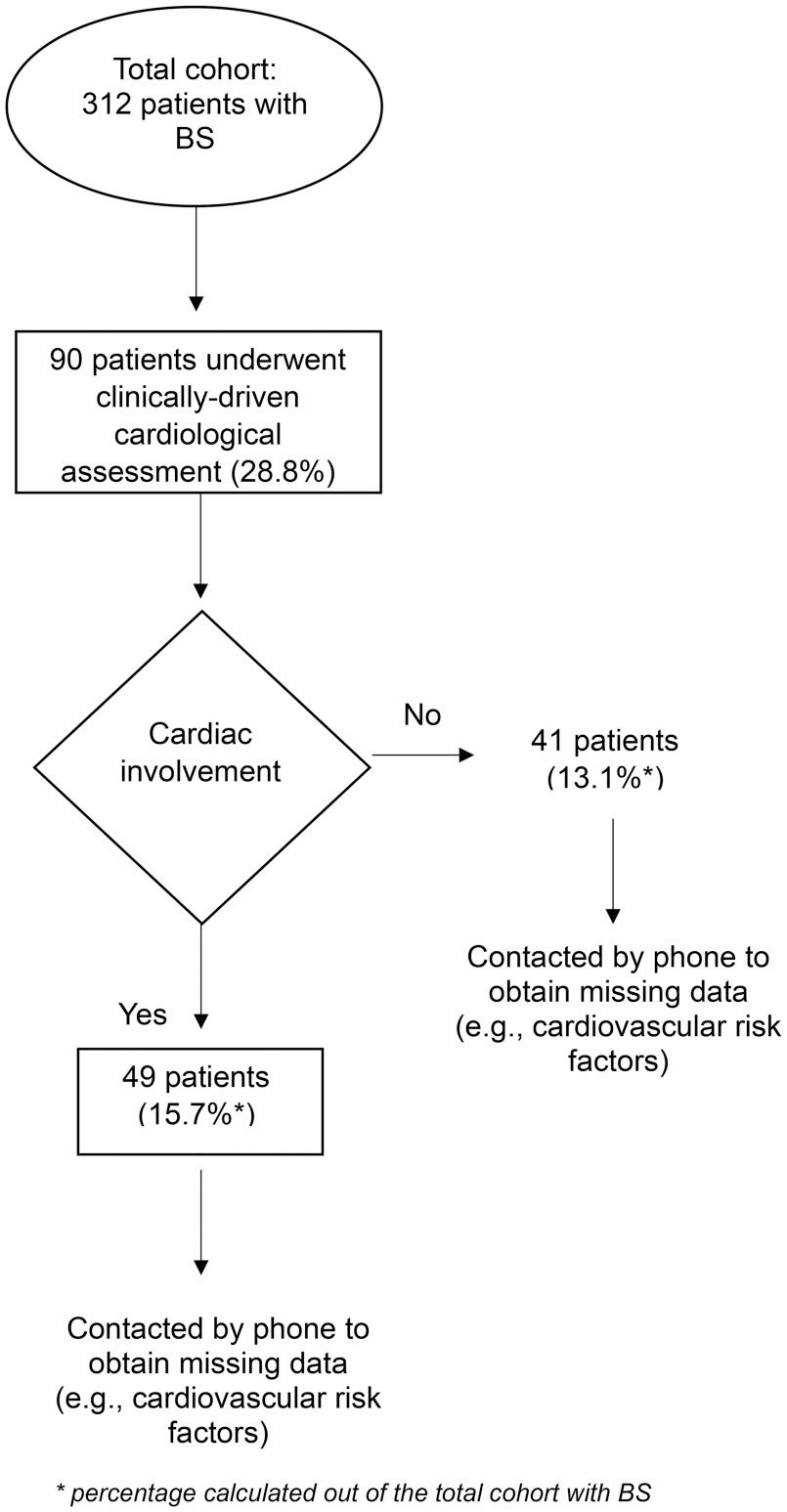
Study flow chart

Out of a total cohort of 312 patients with BS referring to the centre, 90 subjects (28.8%) underwent cardiological assessment: of them, 49 showed a confirmed cardiac involvement (54.4% of patients with cardiological assessment; 15.7% of the whole cohort), whereas the remaining 41 patients had no evidence of cardiac involvement (Fig. 1). The demographic characteristics of patients who underwent cardiological assessment are summarized in Table 1, separately for patients with *vs* those without evidence of cardiac involvement.

**Table 1:** Demographic features and cardiovascular risk profile.

	Cases with cardiac involvement (*n* = 49)	Cases without cardiac involvement (*n* = 41)	*P*-value
Demographic features			
Sex, female, *n* (%)	29 (59.2)	22 (53.7)	0.671
Age at diagnosis, years	39.7 (30.7–44.4)	40.5 (29.9–49.9)	0.444
Age at cardiac assessment, years	49.5 (40.1–57.0)	51.3 (39.1–57.4)	0.779
Age at first cardiac event, years	46.0 (35.1–52.2)		
HLA-B51 positivity, *n* (%)	32 (65.3)	20/38 (52.6)	0.274
Cardiovascular risk profile, *n* (%)			
At least one risk factor	41 (83.7)	28 (68.3)	0.132
BMI ≥25 kg/m^2^	23 (46.9)	13/19^a^ (68.4)	
Hypertension	17 (34.6)	15 (35.6)	
Smoke	15 (30.6)	10 (24.4)	
Family history	12 (24.5)	4 (16.4)	
Dyslipidaemia	11 (22.4)	13 (31.7)	
Diabetes	2 (4.1)	2 (4.9)	
aPL positivity	2 (4.1)	1 (2.4)	
Previous vascular event, *n* (%)	20 (40.8)	22 (53.7)	0.290

Data are presented as absolute frequencies and percentage, or as median value and interquartile range. Exact *P*-values are calculated from the Fisher's test or Mann-Whitney test.

a Data were available for 19 patients of the cohort.

Most patients with cardiac involvement were female (*n* = 29, 59.2%), with a median age at first cardiac event of 46.0 years (IQR 35.1–52.2). HLA-B51 positivity was reported in 32 patients (65.3%), as compared with 20 (52.6%) patients without cardiac involvement (*P* = 0.274).

### Cardiovascular risk profile

Forty-one patients (83.7%) with cardiac involvement had at least one established cardiovascular risk factor, as compared with 28 patients without cardiac involvement (68.3%) (*P* = 0.132). Overweight was the most frequent risk factor, reported in 23 patients with cardiac involvement (46.9%), and six patients were obese (BMI ≥30 kg/m^2^). Other common risk factors included arterial hypertension (*n* = 17; 34.6%), smoking (*n* = 15; 30.6%), family history of cardiovascular disease (*n* = 12; 24.5%) and dyslipidaemia (*n* = 11; 22.4%).

### Previous vascular involvement

Twenty patients (40.8%) with cardiac involvement had history of a previous vascular event, as compared with 22 (53.7%) patients without cardiac involvement (*P* = 0.290).

In patients with cardiac involvement, the vascular event preceded the cardiac one by 3.4 years (IQR 1.1–13.7). Vascular manifestations mainly consisted of superficial (*n* = 7) or deep vein thrombosis (*n* = 12), including three cases of thrombosis at atypical sites (splenic, portal and ovarian veins). Three patients had a prior history of stroke or transient ischaemic attack, two of pulmonary embolism and one of peripheral arterial involvement.

### Cardiac involvement

**Figure 2:**
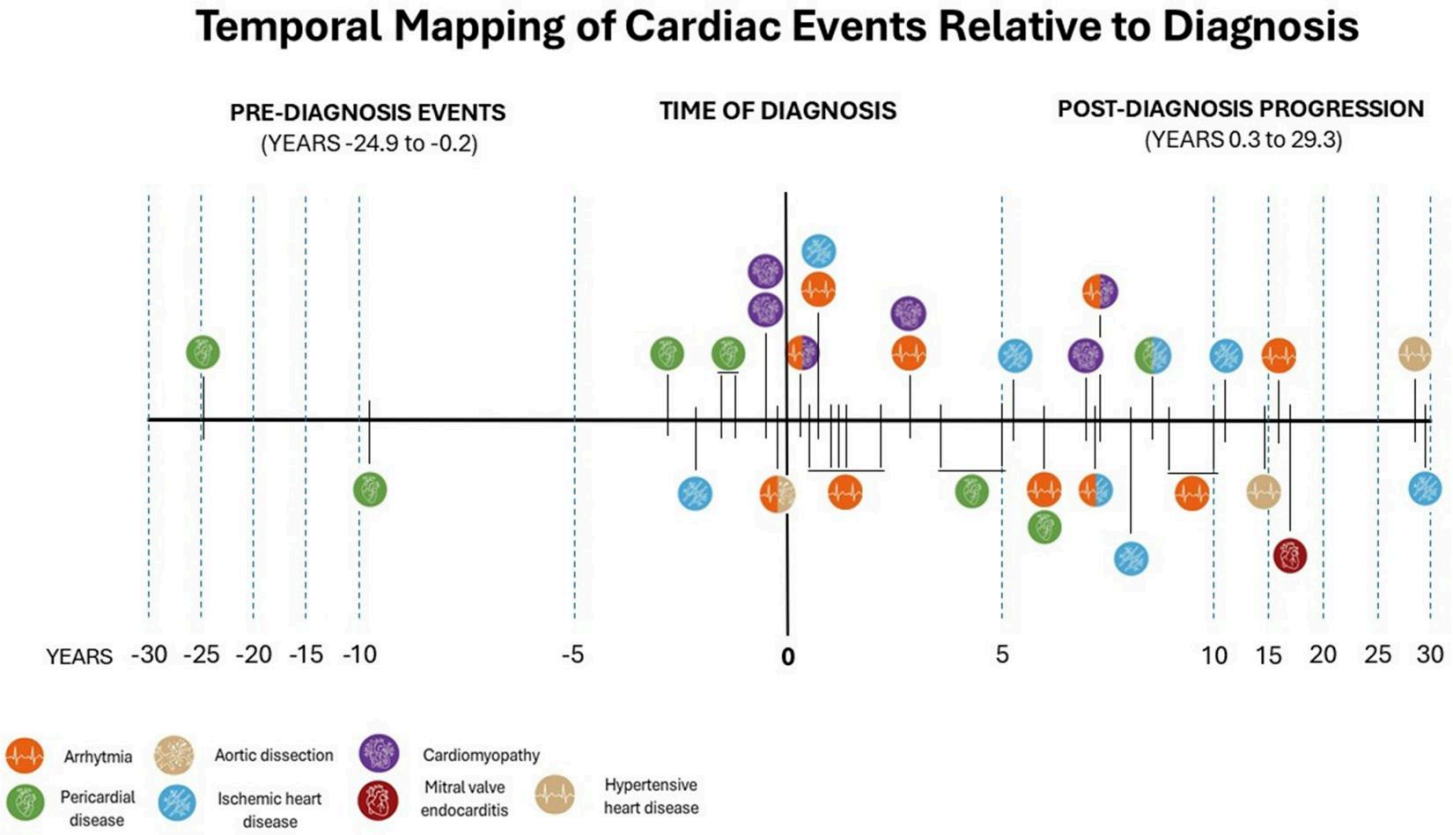
Temporal association of cardiac involvement and Behçet’s syndrome diagnosis. Each symbol represents a cardiac event, classified according to phenotype. The vertical lines indicate the timing of the events, with respect to Behçet’s syndrome diagnosis (vertical line in bold; time 0)

In 40 patients, cardiac involvement occurred after a median time of 6.1 years (2.5–8.7) after the diagnosis of BS (Fig. 2). In six other patients, cardiac involvement preceded the diagnosis of BS of 2.5 years (1.7–8), while in three patients it occurred at the time of diagnosis.

**Figure 3:**
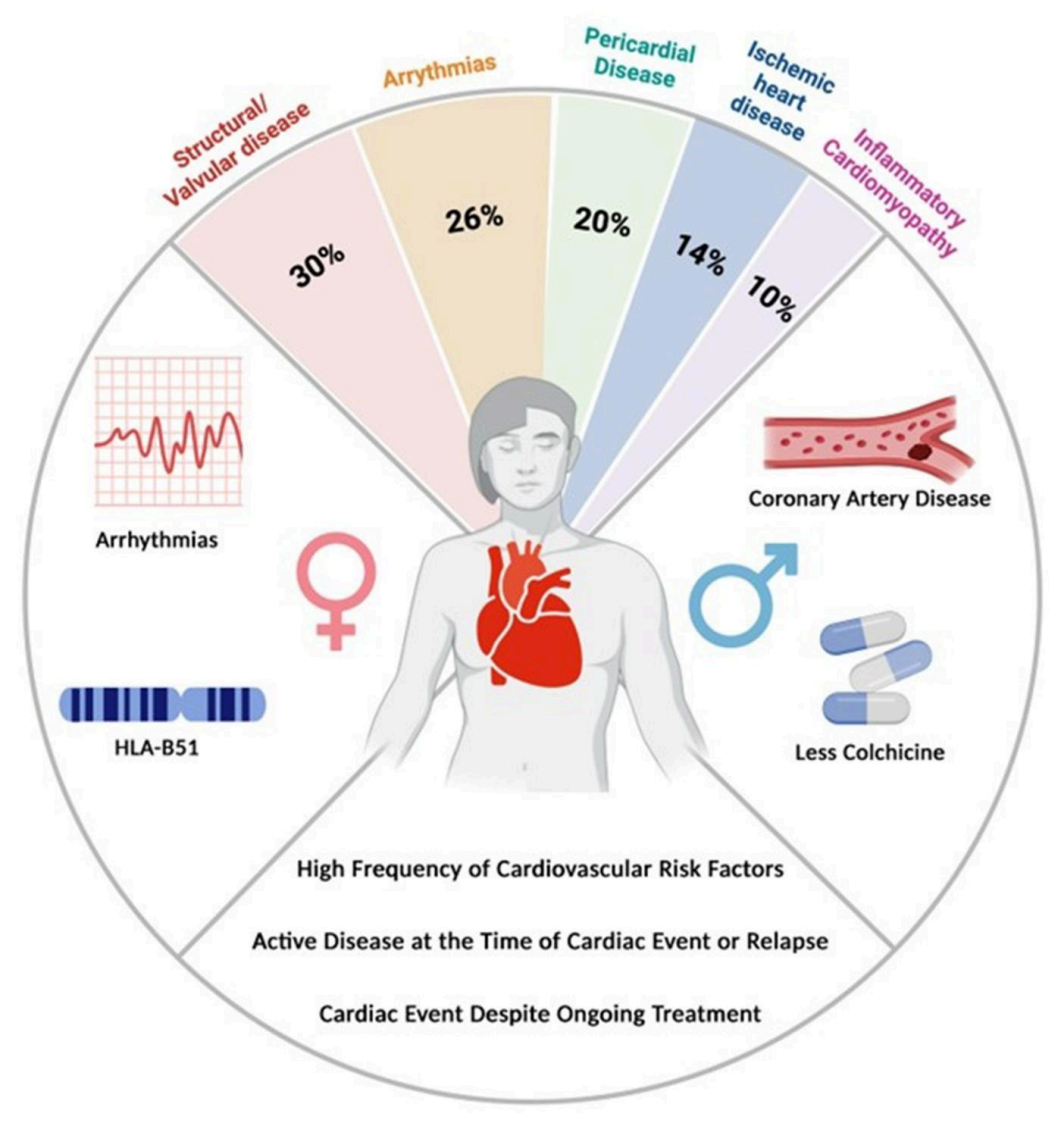
Distribution of cardiac involvement and associated features in Behçet’s syndrome

Arrhythmias were the most frequent cardiac involvement (*n* = 15), mainly presenting as paroxysmal supraventricular tachycardia (*n* = 7) or ventricular extrasystoles (*n* = 4). Pericarditis was the second most common manifestation (*n* = 12), with recurrent pericarditis occurring in half of the cases (*n* = 6). Ischaemic events were reported in eight patients, including three non-ST-segment elevation myocardial infarction (NSTEMI) and three ST-segment elevation myocardial infarction (STEMI). Valvular disease and structural cardiac abnormalities were identified in nine patients each (Fig. 3).

Overall, no deaths attributable to cardiac involvement were recorded.

Supplementary Table S1 reports cardiac involvement, stratified according to sex, previous exposure to colchicine treatment, HLA-B51 positivity and previous vascular involvement.

Arrhythmias tended to be more common in females (*n* = 11/29 females *vs* 4/20 males; 37.9% *vs* 20.0%) and in HLA-B51-positive patients (*n* = 12/32 in HLA-B51 positive *vs* 3/17 in HLA-B51 negative; 37.5% *vs* 17.6%), whereas pericardial disease was more frequently reported in HLA-B51-negative patients (*n* = 6/17 in HLA-B51 positive *vs* 6/32 in HLA-B51 negative; 35.3% *vs* 18.8%).

Ischaemic heart disease tended to be more frequent in male patients (*n* = 6/20 males *vs* 2/29 females; 30.0% *vs* 6.9%) and in patients with previous vascular involvement (*n* = 3/29 patients without *vs* 5/20 with vascular involvement; 10.3% *vs* 25.0%), while it was less frequently reported in patients treated with colchicine (*n* = 6/25 patients not treated *vs* 2/24 treated with colchicine; 24.0% *vs* 8.3%).

### Disease manifestations and treatments

BS manifestations and pharmacological therapies are reported in Tables 2 and 3, separately for patients with and without cardiac involvement.

**Table 2:** Clinical manifestations of Behçet’s syndrome with and without cardiac involvement.

	Cases without cardiac involvement	**Cases with cardiac involvement**			
	Overall (*n* = 41)	Overall (*n* = 49)	***P*-value** ^a^	At time of first event (*n* = 49)	At time of cardiac relapse (*n* = 11)
Active systemic disease				46 (93.9)	11 (100)
Oral aphtosis	40 (95.6)	48 (98.0)	1.000	38 (77.6)	11 (100)
Skin manifestations	27 (65.9)	37 (75.5)	0.356	22 (44.9)	7 (63.6)
Articular involvement	25 (61.0)	34 (69.4)	0.505	25 (51.0)	5 (45.5)
Intestinal involvement	18 (43.9)	32 (65.3)	0.056	20 (41.7)	6 (54.5)
Genital aphtosis	23 (56.1)	28 (57.1)	1.000	10 (20.4)	2 (18.2)
Neurologial manifestations	15 (36.6)	20 (41.7)	0.828	8 (16.3)	0
Vascular lesions	22 (53.7)	20 (40.8)	0.299	8 (16.3)	3 (27.3)
Ocular lesions	16 (39.0)	12 (24.5)	0.172	8 (16.3)	1 (9.1)
Positive pathergy test	5 (12.2)	12 (24.5)	0.180		

Data are presented as *n* (%).

a *P*-value for the comparison of patients with *vs* without evidence of cardiac involvement, using Fisher’s exact test.

**Table 3:** Pharmacological treatments in patients with Behçet’s syndrome.

Treatments	Cases without cardiac involvement (*n* = 41)	Cases with cardiac involvement (*n* = 49)			
		Before cardiac involvement	***P*-value** ^a^	At time of first event	At time of cardiac relapse (*n* = 11)
Prednisone	30 (73.2)	32 (65.3)	0.496	25 (51.0)	7 (63.6)
Daily dose, mg, median (IQR)				5 (5–9)	5 (5–9)
Colchicine	22 (53.7)	24 (49.0)	0.678	17 (34.7)	6 (54.6)
Traditional DMARD treatment	25 (61.0)	28 (57.1)	0.830		
MTX	12 (29.3)	10 (20.4)		4 (8.2)	3 (27.3)
Ciclosporin	6 (14.6)	6 (12.2)		2 (4.1)	0
AZA	17 (41.5)	21 (42.9)		13 (26.5)	3 (27.3)
Other	3 (1 CYC) (7.3)	5 (1 CYC) (10.2)		0	2 (1 CYC; 1 MMF) (18.2)
IFN	2 (4.9)	1 (2.0)		1 (2.0)	0
Biologic DMARD treatment	23 (56.1)	29 (59.2)	0.832		
Etanercept	4 (9.8)	5 (10.2)		1 (2.0)	0
Infliximab	5 (12.2)	8 (16.3)		5 (10.2)	1 (9.1)
Adalimumab	16 (39.0)	19 (38.8)		10 (20.4)	5 (45.5)
Golimumab	9 (22.0)	6 (12.2)		4 (8.2)	0
Certolizumab	3 (7.3)	2 (4.1)		2 (4.1)	0
Secukinumab	4 (9.8)	4 (8.2)		3 (6.1)	1 (9.1)
Ustekinumab	2 (4.9)	0		0	0
Anakinra	1 (2.4)	3 (6.1)		0	1 (9.1)
Canakinumab	1 (2.4)	1 (2.0)		1 (2.0)	0
Other drugs	3 (7.3)	2 (4.1)		1 (2.0)	2 (18.2)
Cardiovascular treatment	24 (58.5)	19 (38.8)	0.090	After the first event	Started after the first event
Antiplatelet drugs	8 (19.5)	5 (10.2)		15 (30.6)	2 (18.2)
Anticoagulants	8 (19.5)	6 (12.2)		8 (16.3)	0
Antiarrhythmic drugs	8 (19.5)	3 (6.1)		18 (36.7)	3 (27.3)
Antihypertensive drugs	15 (36.6)	10 (20.4)		15 (30.6)	2 (18.2)
Lipid-lowering drugs	6 (14.6)	2 (4.1)		11 (22.5)	1 (9.1)

Data are presented as *n* (%) unless otherwise stated.

a *P*-value for the comparison of patients with *vs* without evidence of cardiac involvement.

Regarding BS manifestations recorded in the whole medical history, almost all patients with cardiac involvement presented oral mucosal involvement (*n* = 48; 98.0%), often accompanied by cutaneous (*n* = 37; 75.5%), articular (*n* = 34; 69.4%), intestinal (*n* = 32; 65.3%) and genital mucosal involvement (*n* = 28; 57.1%). Ocular involvement and a positive pathergy test were each observed in 12 patients (24.5%).

Overall disease manifestations were similar in patients with and without cardiac involvement, with the exception of intestinal involvement, which seemed to be less common in patients without cardiac involvement (*n* = 18/41; 43.9%) (*P* = 0.056).

At the time of first cardiac event, all except three patients presented with active disease (93.9%), defined as the presence of at least one active manifestation besides cardiac involvement. Specifically, a relevant proportion of patients presented active oral manifestations (*n* = 38, 77.6%), and active cutaneous or articular involvement was reported in 45–51% of patients.

Overall exposure to traditional and biologic DMARDs appeared comparable in patients with and without cardiac involvement. Cardiac involvement occurred on active pharmacological treatment in 41/49 patients (83.7%), with glucocorticoids in 25 patients (51%), biologic DMARDs in 27 (55.1%) and traditional DMARDs in 17 (34.7%) (Table 3).

Before the cardiac involvement, only a minority of patients were receiving cardiovascular preventive therapy (38.8%): antihypertensive agents (*n* = 10; 20.4%), anticoagulants (6; 12.2%), antiplatelet agents (5; 10.2%), antiarrhythmic drugs (3; 6.1%) and lipid-lowering therapy (2; 4.1%). As expected, the use of these medications increased after the first cardiac event (65.3%), ranging from 16.3% for anticoagulants to 36.7% for antiarrhythmic agents. However, the use of cardiovascular preventive therapy appeared more frequent in patients without cardiac involvement, particularly the use of antiarrhythmic drugs (*n* = 15/41; 19.5%) and lipid-lowering agents (*n* = 6/41; 14.6%).

### Cardiac relapse

Eleven patients with cardiac involvement experienced a cardiac relapse, and another three a vascular relapse (deep vein thrombosis).

Cardiac relapses included eight cases of pericarditis (including one myopericarditis), six of which occurred in patients with previous pericarditis; one NSTEMI in a patient with prior STEMI; one episode of atrial fibrillation in a patient with previous myocarditis; and one recurrence of ventricular extrasystoles.

At the time of cardiac relapse, all patients had active mucosal manifestations, mostly associated with active cutaneous (*n* = 7; 63.6%), intestinal (6; 54.5%) or articular (5; 45.5%) involvement (Table 2).

Cardiac relapse occurred during ongoing glucocorticoid therapy in 7 out of 11 cases (63.6%), while 6 patients were receiving traditional DMARDs (54.6%) and 8 biologic DMARDs (72.7%). Only a minor proportion of these patients were receiving active cardiovascular prophylaxis, including antiarrhythmic drugs (*n* = 3; 27.3%), antiplatelets and antihypertensive agents (*n* = 2 each, 18.2%) and lipid-lowering drugs (*n* = 1).

## Discussion

In this single-centre retrospective clinically driven study, we characterized cardiac involvement in a well-defined cohort of patients with BS who underwent active cardiological evaluation. Our findings highlight that cardiac manifestations, although traditionally considered rare, may be more frequently detected than previously reported in the literature (5–8%) [2, 3], particularly when actively investigated. In this context, we observed a prevalence of cardiac involvement of ∼15% in the whole BS cohort, and exceeding 54% among patients undergoing clinically driven cardiological assessment. Compared with previous reports, our cohort was not passively identified through clinically overt cardiovascular complications recorded in medical charts, but derived from a population in which patients were routinely referred to a cardiological assessment as part of standard clinical practice, either for screening or for suggestive symptoms.

This clinically driven approach focuses on patients referred for suspected cardiac involvement which likely increased the detection of cardiac abnormalities, potentially explaining the higher prevalence observed in this study.

This result supports the notion that cardiac involvement in BS may be remarkably underrecognized in routine care, particularly when cardiological investigations are performed only in the presence of relevant manifestations.

Cardiac involvement occurred at a median of 6 years after BS diagnosis, although in a subset it preceded or coincided with the diagnosis, confirming that cardiac manifestations may represent an early manifestation or even the presentation feature of the disease [2, 3, 6]. Importantly, almost all patients had active systemic disease at the time of the first cardiac event, and all patients with cardiac relapse had concomitant active manifestations. This association reinforces the concept that cardiac involvement in BS is closely linked to the inflammatory and prothrombotic disease activity, rather than being merely the consequence of traditional cardiovascular risk factors. In addition, it must be noted that >80% of patients were on active immunosuppressant/immunomodulating treatment at the time of cardiac involvement, with >50% being on biologic DMARDs, likely reflecting the underlying severity of disease.

On the other hand, a striking finding is the apparent underuse of cardiovascular preventive therapies. Before cardiac involvement, <40% of patients were receiving antihypertensive agents, antiplatelet therapy, anticoagulation or lipid-lowering drugs, despite a high burden of traditional cardiovascular risk factors, with over 80% of patients with at least one established risk factor. Even after the first cardiac event, although the use of these medications increased, the overall proportion of patients receiving guideline-directed preventive therapy remained suboptimal (65.3%). Given the observational nature of this study and the absence of detailed information on clinical indications or treatment-decision processes, we can only speculate about the reasons underlying this discrepancy. Namely, this undertreatment may reflect several factors, including the relatively young age of the population, the attribution of cardiac involvement primarily to inflammatory mechanisms rather than atherosclerotic disease, the established role of immunosuppression in controlling thromboinflammation [15, 16], or fragmentation between rheumatologic and cardiologic care. Overall, our findings suggest the need for an integrated cardio-rheumatologic approach in BS, combining control of systemic inflammation with appropriate management of modifiable cardiovascular risk factors.

Regarding the type of cardiac involvement, arrhythmias emerged as the most frequent cardiac manifestation in our cohort (30.6%), mostly related to paroxysmal supraventricular tachycardia and ventricular extrasystoles. The attribution of cardiac manifestations to inflammatory mechanisms may be complex. While some conditions are more clearly inflammatory, others, such as ischaemic events and arrhythmias, may also be influenced by traditional cardiovascular risk factors and, particularly for milder arrhythmic findings, may partly reflect incidental detection in a clinically driven evaluation setting. However, the strong association observed with disease activity suggests that systemic inflammation may, at least in part, contribute also to manifestations that are traditionally considered less directly inflammatory. Arrhythmias are uncommonly reported in patients with BS [6], but studies assessing the arrhythmogenic potential of BS reported an increased ventricular dispersion (i.e. the difference between the longest and the shortest QT intervals within a 12-lead ECG) in cases with BS as compared with controls [17–21], indicating an increased risk of ventricular arrhythmias in BS. More recently, a study assessing ventricular and atrial function in patients with BS did not find any deterioration in left or right ventricular function in BS, but reported a significantly lower left atrium strain reservoir in BS patients as compared with the control group [22]. Accordingly, literature data indicate an ∼1.8 higher risk of atrial fibrillation in BS as compared with the general population, likely attributable to chronic inflammation and atrial structural remodelling [23]. Conversely, within our cohort only two patients reported atrial fibrillation.

Sex-related differences also emerged from our analysis. Women represented 59% of the whole cohort, and more frequently experiences arrhythmias as compared with men (38% *vs* 20%). On the other hand, male patients tended to experience more severe cardiac manifestations, particularly ischaemic heart disease (30% *vs* 2% among men and women). This observation is consistent with the well-established association between male sex and more severe disease.

Also, HLA-B51 positivity was associated with a higher prevalence of arrhythmias in our cohort (38%, as compared with 18% in HLA-B51-negative patients). These data parallel previous reports linking another MHC class I allele—HLA-B27—to cardiac conduction abnormalities, atrioventricular block and other electrophysiological abnormalities [24–28], although contrasting results have been published [29]. The structural and functional similarities between HLA-B27-associated cardiac involvement and the arrhythmic manifestations observed in our cohort raise the possibility that similar, while still largely unknown, immunopathogenic pathways may be involved in HLA-B51-positive patients with BS. Although direct evidence linking HLA-B51 to arrhythmic complications is currently limited, our findings support the hypothesis that genetic background may influence the pattern of cardiac involvement in BS.

Contrary to what might be expected, in our cohort the prevalence of previous vascular events was comparable between patients with and without cardiac involvement at the time of cardiological assessment. This finding may appear counterintuitive, as vascular involvement is linked to a higher inflammatory state, and could potentially predispose to a higher risk of cardiac complications. However, this observation is likely explained by the clinically driven nature of cardiological referrals in our cohort. Thus, it is plausible that patients with documented vascular involvement were commonly referred for a more comprehensive cardiovascular evaluation, and hence, the prevalence of prior vascular events was high also in the non-cardiological group.

Despite the occurrence of clinically significant events, including STEMI, NSTEMI and one cardiac arrest, no deaths attributable to cardiac involvement were recorded in our cohort. This result contrasts literature data reporting a 5-year mortality of up to 16% in patients with cardiac involvement [2], but represents a reassuring finding, which may reflect timely recognition, access to specialized care and the generally favourable short- to mid-term prognosis of cardiac involvement when appropriately managed in a tertiary centre setting.

Our study has limitations inherent to its retrospective design and single-centre setting, including potential referral bias, limited statistical power for solid inferential analyses and missing data (e.g. BMI) for a subset of patients. Also, data on cardiovascular risk factors were collected by patient phone call, and recall bias cannot be excluded.

On the other hand, the clinically driven review of medical records and the availability of detailed longitudinal data allowed us to comprehensively characterize the timing, phenotype and management of cardiac involvement in a well-defined BS cohort.

In conclusion, in a cohort of patients with BS undergoing active cardiological assessment, cardiac involvement was more frequent than traditionally reported. Female sex was more common among patients with cardiac involvement, yet male patients tended to experience more severe events, particularly ischaemic manifestations. Overall, no cardiac-related deaths were observed. The high prevalence of cardiovascular risk factors, combined with the suboptimal use of preventive therapies even after cardiac involvement, underscores the need for structured cardiovascular risk assessment in patients with BS, integrating rheumatologic and cardiologic care.

## Supplementary Material

keag292_Supplementary_Data

## Data Availability

Deidentified participant data are available upon reasonable request, to be sent by email to the corresponding author.
