# Supplementary material for: Cardiac involvement in Behçet’s syndrome: findings from a clinically driven cardiological evaluation
Source: Rheumatology (Oxford). 2026 Jul 2;65(7):keag292. doi: 10.1093/rheumatology/keag292 (PMC13332428; doi:10.1093/rheumatology/keag292)
Supplement: keag292_Supplementary_Data [file keag292_supplementary_data.docx]

**SUPPLEMENTARY MATERIAL**

**Supplementary Table S1.** Detail of cardiac involvement in the cohort, stratified according to sex, use of colchicine and HLA-B51 positivity

|  | **Overall** | **Sex** | | **Colchicine** | | **HLA-B51** | | **Previous vascular involvement** | |
| --- | --- | --- | --- | --- | --- | --- | --- | --- | --- |
|  | **N cases** | **Males**  **(n=20)** | **Females (n=29)** | **No**  **(n=25)** | **Yes**  **(n=24)** | **Negative**  **(n=17)** | **Positive**  **(n=32)** | **No**  **(n=29)** | **Yes**  **(n=20)** |
| **Arrhythmias** | **15** | **4** **(20.0%)** | **11 (37.9%)** | **8 (32.0%)** | **7 (29.2%)** | **3 (17.6%)** | **12 (37.5%)** | **8 (27.6%)** | **7 (35.0%)** |
| PSVT | 7 | 1 | 6 | 4 | 3 | 2 | 5 | 2 | 5 |
| Atrial fibrillation | 2 | 1 | 1 | 1 | 1 | 0 | 2 | 2 |  |
| Ventricular extrasystoles | 4 | 1 | 3 | 2 | 2 | 1 | 3 | 3 | 1 |
| Sinus tachycardia + conduction disorder | 1 |  | 1 |  | 1 |  | 1 | 1 |  |
| Cardiac arrest | 1 | 1 |  | 1 |  |  | 1 |  | 1 |
| **Pericardial disease** | **12** | **4 (20.0%)** | **8 (27.6%)** | **5 (20.0%)** | **7 (29.2%)** | **6 (35.3%)** | **6** **(18.8%)** | **6 (20.7%)** | **6 (30.0%)** |
| **Acute pericarditis** | 4 | 2 | 2 | 2 | 2 | 3 | 1 |  | 4 |
| **Recurrent pericarditis** | 6 | 1 | 5 | 2 | 4 | 2 | 4 | 5 | 1 |
| **Pericarditis with pericardial effusion** | 1 |  | 1 |  | 1 |  | 1 | 1 |  |
| **Isolated pericardial effusion** | 1 | 1 |  | 1 |  | 1 |  |  | 1 |
| **Ischemic heart disease** | **8** | **6 (30.0%)** | **2 (6.9%)** | **6 (24.0%)** | **2 (8.3%)** | **2 (11.8%)** | **6 (18.8%)** | **3 (10.3%)** | **5 (25.0%)** |
| NSTEMI | 3 | 2 | 1 | 2 | 1 |  | 3 | 1 | 2 |
| STEMI | 3 | 2 | 1 | 3 |  | 1 | 2 | 1 | 2 |
| Old/undiagnosed MI | 1 | 1 |  | 1 |  | 1 |  |  | 1 |
| Chronic coronary artery disease with stable angina | 1 | 1 |  |  | 1 |  | 1 | 1 |  |
| **Structural heart disease** | **8** | **3 (15.0%)** | **5 (17.2%)** | **4 (16.0%)** | **4 (16.7%)** | **2 (11.8%)** | **6 (18.8%)** | **5 (17.2%)** | **3 (15.0%)** |
| Patent foramen ovale | 8 (closed in 3) | 3 | 5 | 4 | 4 | 2 | 6 | 5 | 3 |
| Atrial septal aneurysm | 3 |  | 3 | 1 | 2 |  | 3 | 3 |  |
| **Valvular disease** | **9** | **3** | **6** | **5** | **4** | **2** | **7** | **6 (20.7%)** | **3 (15.0%)** |
| Mitral valve disease | 7 | 3 | 4 | 4 | 3 | 1 | 6 | 5 | 2 |
| Tricuspid regurgitation | 3 |  | 3 | 1 | 2 | 1 | 2 | 2 | 1 |
| **Myocardial / inflammatory cardiomyopathies** | **6** | **2 (10.0%)** | **4 (13.8%)** | **3 (12.0%)** | **3 (12.5%)** | **2 (11.8%)** | **4 (12.5%)** | **4 (13.8%)** | **2 (10.0%)** |
| Myocarditis | 4 | 1 | 3 | 2 | 2 |  | 4 | 2 | 2 |
| **Takotsubo syndrome** | 1 |  | 1 | 1 |  | 1 |  | 1 |  |
| Suspected cardiac amyloidosis | 1 | 1 |  |  | 1 | 1 |  | 1 |  |
| Hypertensive heart disease | 2 | 1 | 1 |  | 2 | 1 | 1 | 1 |  |
| Pulmonary hypertension | 1 | 1 |  | 1 |  |  | 1 |  | 1 |
| Aortic dissection | 1 |  | 1 |  | 1 |  | 1 |  | 1 |
| **Mitral valve endocarditis** | 1 | 1 |  |  | 1 | 1 |  |  | 1 |
